# Relevance of Nutrient-Sensing in the Pathogenesis of *Trichophyton rubrum* and *Trichophyton interdigitale*

**DOI:** 10.3389/ffunb.2022.858968

**Published:** 2022-03-31

**Authors:** Aline H. S. Cruz, Rodrigo S. Santos, Maíra P. Martins, Nalu T. A. Peres, Glauce L. Trevisan, Niege S. Mendes, Nilce M. Martinez-Rossi, Antonio Rossi

**Affiliations:** ^1^Department of Genetics, Ribeirão Preto Medical School, University of São Paulo, Ribeirão Preto, Brazil; ^2^Department of Biochemistry and Molecular Biology, Institute of Biological Sciences, Federal University of Goiás, Goiânia, Brazil; ^3^Department of Microbiology, Institute of Biological Sciences, Federal University of Minas Gerais, Belo Horizonte, Brazil

**Keywords:** *Trichophyton*, tricarboxylic acid cycle, glyoxylate cycle, methylcitrate cycle, cellular cycle, subtilisin

## Abstract

The growth and development of organisms depend on nutrient availability. Dermatophytes must sense nutrient levels and adapt to the host environment to colonize human and animal keratinized tissues. Owing to the clinical importance of the *Trichophyton* genus, this study compared the expression profile of genes involved in metabolism, cell cycle control, and proteases in two *Trichophyton* species, *Trichophyton rubrum*, and *Trichophyton interdigitale*, in response to nutrients and environmental pH. In addition, we evaluated the activity of enzymes in the tricarboxylic acid, glyoxylate, and methylcitrate cycles. Moreover, the effects of interruption of the transcription factor *pacC* on *T. interdigitale* in the same conditions as for the wild-type strain were determined. Our analyses revealed specific responses in each species to the nutritional and pH variation. An improved adaptation of *T. interdigitale* to keratin was observed, compared with that of *T. rubrum*. *T. rubrum* growth in buffered keratin media indicated pH 8.0 as an optimal pH condition for metabolic activity, which differed from that for *T. interdigitale*. Tricarboxylic acid components in *T. rubrum* showed increased enzymatic activity and transcript accumulation. In *T. interdigitale*, a higher activity of enzymes in glyoxylate and methylcitrate cycles was observed, with no direct correlation to the transcriptional profile. *T. interdigitale* fungal metabolism suggests the requirement of anaplerotic pathways in the late cultivation period. The identified differences between *T. rubrum* and *T. interdigitale* may represent determinants for adaptation to the host and the incidence of infection with each species.

## Introduction

Nutrient sensing and signaling modulate cell growth in various organisms. The ability to use different nutrition sources is crucial in pathogenic fungi, given that they must acquire nutrients from different host tissues. Dermatophytes are fungi that use keratin as a nutrient source, thereby infecting different niches, such as human and animal tissues (skin, hair, and nails), consequently causing cutaneous diseases (Grumbt et al., 2011; Gnat et al., 2019). *Trichophyton rubrum* and *Trichophyton interdigitale* are anthropophilic and cosmopolitan dermatophytes. They cause the diseases tinea corporis and tinea pedis, and represent two major pathogens that cause tinea unguium (Mehul et al., 2019; Martinez-Rossi et al., 2021).

Data on the molecular aspects of nutrient acquisition and cellular growth in dermatophytes remain scarce. Different genes are expressed in response to *in vitro, ex vivo*, or *in vivo* infection models (Zaugg et al., 2008; Peres et al., 2016; Tran et al., 2016). Additionally, *in vitro* analysis have revealed that during the growth and development of *Trichophyton* in glycine, keratin, and lipids as a carbon source, the extracellular pH shifts from acidic to alkaline due to the secretion of ammonia (Ferreira-Nozawa et al., 2003; Maranhão et al., 2007, 2011; Silveira et al., 2010; Mendes et al., 2012) and urea (Martins et al., 2020). Furthermore, the alkalinization of the culture medium by *T. interdigitale* when grown in keratin upregulates genes encoding subtilisins and metalloproteases (Maranhão et al., 2007), whereas acidic conditions regulate the expression of genes associated with the glyoxylate cycle (GC) and heat shock proteins (Peres et al., 2010). It was concluded that extracellular pH changes are necessary for regulating gene expression as an efficient strategy for colonization, development, and maintenance in the host tissue (Martinez-Rossi et al., 2012, 2017).

The highly conserved signal transducer pathway involved in the adaptive response to pH comprises at least six *pal* genes (*palA, palB, palC, palF, palH*, and *palI*). In many filamentous fungi, this signaling cascade mediates the proteolytic processing of the transcription factor PacC, which is functional in acidic and alkaline environments (Martinez-Rossi et al., 2012; Rossi et al., 2013; Li et al., 2021). A Δ*pacC* strain of *T. interdigitale* (formerly *T. rubrum*), which contains the disrupted *pacC* gene, shows reduced growth in nail fragments and the decreased secretion of keratinolytic proteases, directly associating this transcription factor to the proteolytic activity of *T. interdigitale* H6 (Ferreira-Nozawa et al., 2006).

In this study, we assessed the expression profile of genes involved in metabolism (*idh1, idh2, idhp, icl*, and *meicl*), cell cycle control (*mad2* and *mad2B*), and proteolytic activity (*sub3* and *sub5*) in the *Trichophyton* species, *T. rubrum* and *T. interdigitale* along with the *T. interdigitale pacC* mutant strain, in response to nutrient source and environmental pH. In addition, we evaluated the enzymatic activity and phosphorylation to explore their influence on fungal development. We further explored the effects of *pacC* deletion on *T. interdigitale* adaptation to pH variation, highlighting the relevance of this transcription factor in dermatophyte adaptation. Considering the high incidence of infections caused by dermatophytes, which affects nearly 25% of the world's population (Martinez-Rossi et al., 2021), especially by *Trichophyton* species, we aimed to increase the knowledge of the molecular mechanisms of these pathogens associated with nutrient acquisition, growth, and survival.

## Materials and Methods

### *Trichophyton* Strains

The strains used in this study included *T. rubrum* strain CBS 118892 (Westerdijk Fungal Biodiversity Institute, formerly CBS-KNAW, Utrecht, Netherlands); *T. interdigitale* H6 strain (ATCC MYA-3108) isolated at the University Hospital of Ribeirão Preto Medical School, São Paulo University, Brazil, and the Δ*pacC* strain obtained from *T. interdigitale* H6, which carries a disrupted *pacC* gene (Ferreira-Nozawa et al., 2006). The H6 strain was previously classified as *T. rubrum* and reclassified as *T. interdigitale* (Persinoti et al., 2018).

### Fungal Culture Conditions

The strains were cultured on malt extract agar (pH 5.7) at 28°C. Conidia suspensions were obtained by flooding 22-day-old plates with sterilized 0.9% NaCl, recovering the liquid, mixing by vortex, and filtering through glass wool. Conidia concentration was estimated by counting in the Neubauer chamber. For growth in the liquid medium, 1 × 10^7^ conidia from each strain were inoculated into 50 ml of sabouraud dextrose broth (SDB; pH 5.7). The 96 h incubation was performed under continuous shaking at 100 rpm at 28°C. For unbuffered culture conditions, we individually transferred mycelia from a 96 h culture to 100 ml of minimal medium (MM) (Cove, 1966) at pH 5.0, supplemented with 2 mM KH_2_PO_4_ and the following carbon sources: 55 mM glucose, 50 mM glycine, 55 mM glucose plus 50 mM glycine, or 4 g/L powdered ox hull keratin. Cultures were incubated under continuous shaking at 100 rpm for 24, 48, 72, and 96 h. For buffered conditions, the SDB 96 h culture mycelia were transferred to MM adjusted with 50 mM sodium citrate to pH 5.0 or with 50 mM Tris–HCl to pH 8.0. Buffered cultures were incubated for 24 h under continuous shaking. After each culture period, the mycelia were aseptically filtered and the pH of the medium was measured. The mycelium was dried in sterilized filter paper, weighed, frozen in liquid nitrogen, and stored at −80°C until use.

### RNA Isolation, cDNA Synthesis, and Quantitative Real-Time PCR (RT-qPCR)

Total RNA was extracted from frozen mycelium using TRIzol™ reagent (Invitrogen, Carlsbad, CA, USA) following the manufacturer's instructions. The purified RNA from each experimental condition was reverse transcribed to cDNA using the High-Capacity cDNA Reverse Transcription Kit (Applied Biosystems, Foster City, CA, USA) following the manufacturer's instructions. The specific primer pairs for each gene are listed in Table 1. Quantitative real-time PCR (RT-qPCR) was performed in a final volume of 12.5 μl, containing 2.5 μM of each primer, 1 × *SYBR Green* (PCR mix), and 50 ng cDNA. Amplification was performed in the StepOnePlus™ Real-Time PCR system (Applied Biosystems). The PCR cycle consisted of 2 min at 50°C, 10 min at 95°C, and 40 cycles of 95°C for 15 s, and 60°C for 1 min. The melting curve analysis was performed using the Dissociation Curves Software version 1.0 (Applied Biosystems) to exclude primer dimers and unspecific PCR products. We calculated the relative transcript quantity using the ΔΔCt method (Livak and Schmittgen, 2001), with glyceraldehyde-3-phosphate dehydrogenase (*gapdh*, TERG_04402) and beta-tubulin (β*-tub*, TERG_07904) as endogenous reference genes (Jacob et al., 2012).

**Table 1:** List of primers used in RT-qPCR analysis.

**Gene**	**Gene product name**	**ID**	**Primer sequences (5^**′**^– 3^**′**^)**
*idh1*	Isocitratede hydrogenase [NAD] subunit 1, mitochondrial	TERG_01670	F: TAAGGACCAGGCTAACCC
			R: GACGGCTCGGGTGAAC
*idh2*	Isocitratede hydrogenase, NAD-dependent, mitochondrial	TERG_07814	F: GGTTCCGCTCCCGATA
			R:TGTTTTTGATGATGGCGT
*idhp*	Isocitrate dehydrogenase [NADP], mitochondrial	TERG_06075	F: TCCTGAAGAAATACGATGGC
			R:CGGCAGGGGTGGTCA
*icl*	Isocitrate lyase	TERG_00825	F: ACCGACTTGTAGCCATCC
			R: GTTCTTGCCCGCTTGCTCT
*meicl*	Mitochondrial 2-methylisocitrate lyase (*Trichophyton tonsurans*)	TERG_01271	F: CGCAGAGATTGATGTTTACG
			R: GGGTTCTTCGTGTATTTGG
*mad2*	Mitotic spindle checkpoint protein *mad2* (*Trichophyton equinum*)	TERG_03823	F: TGAAAACGCCAATCCG
			R: CCATTCCAGAGGCACTTC
*mad2B*	Mitotic spindle checkpoint protein *mad2b* (*Trichophyton benhamiae*)	TERG_02307	F: CGAGCCATTGACGCAG
			R: CGCAGTTTCTTCCACACAG
*sub3*	Subtilisin-like protease 3	TERG_03815	F: CTGGTATCTTCGTGGCTGT
			R: AGCGGAGAGGATGTTGG
*sub5*	Subtilisin-like protease 5	TERG_08201	F: TTTATGCTCCCGGTCACAAT
			R: AGTGGGCTGACTGAGCTGTT
*gapdh*	Glyceraldehyde 3-phosphate dehydrogenase	TERG_04402	F: GCGTGACCCAGCCAACA
			R: CGGTGGACTCGACGATGTAGT
*β-* tub	Tubulin beta chain	TERG_07904	F: CCGTATGATGGCCACTTT
			R: CTGACCTGGGAAACGAAGAC

Data normalization was performed using the GenEx 5 MultiD Analyses AB tool (www.multid.se), and the relative quantification was expressed as 2^−Δ*ΔCt*^, converted to log2. The reference conditions for relative expression displayed the lowest expression in each analysis, converting the resulting values into log2. The statistical analysis of the RT-qPCR data was performed using one-way ANOVA followed by the Bonferroni post-test correction using GraphPad Prism v. 5.01 (GraphPad Software, La Jolla, CA, USA).

### Enzymatic Activity Assays

The mycelia obtained from *T. rubrum* CBS 118892 cultured in SDB for 96 h, and from cultures incubated for an additional 24 and 96 h in MM containing glucose, glycine, glucose plus glycine, or keratin, were collected for enzymatic assays. Furthermore, mycelia were collected from *T. interdigitale* H6 and *pacC* mutant strain of *T. interdigitale* in 96 h SDB cultures, and from 24 and 96 h cultures in MM supplemented with keratin. The mycelium was frozen and macerated in liquid nitrogen until a fine powder was obtained to analyze intracellular enzyme activities. The macerate was transferred to 50 ml Falcon tubes and cooled on ice. To 0.75 g of each macerated mycelium, 500 μl of Tris-HCl buffer (50 mM Tris-HCl, 2 mM MgCl_2_, 2 mM dithiothreitol, pH 8.0) was added. Samples were vortexed, dispensed in 2 ml tubes, and centrifuged for 30 min at 1,268 × g at 4°C. The supernatant (protein extract) was collected and kept on ice for use in the enzymatic assays. Proteins were quantified using Bradford reagent (Sigma-Aldrich, St. Louis, MO, USA) according to the manufacturer's instructions.

### Intracellular Enzyme Assay

Isocitrate lyase (ICL) activity was determined using a phenylhydrazine-based assay (Ebel et al., 2006). The reaction was initiated by adding the isocitrate substrate and the glyoxylate phenylhydrazone product was determined at 324 nm (ε 16.8 mM^−1^ cm^−1^; Brock et al., 2001). Under the assessed conditions, one enzyme unit was defined as the amount of enzyme that produced 1 mmol of glyoxylate phenylhydrazone per minute. Specific activity was established as units per milligram (U mg^−1^) of protein used in the experiment.

Isocitrate lyase activity associated with protein dephosphorylation was measured at 96 h of cultivation. The assays were performed using FastAP™ Thermosensitive Alkaline Phosphatase (Thermo Fisher Scientific, Cleveland, OH, USA) by following the manufacturer's instructions. The supernatant (protein extract) was evaluated before and immediately after treatment with FastAP™.

The methylisocitrate lyase (MeICL) activity was determined based on the formation of phenylhydrazone pyruvate (Brock et al., 2001). The reaction was initiated with the addition of methylisocitrate substrate. The formation of phenylhydrazone pyruvate product was determined at 324 nm (ε 16.8 mM^−1^ cm^−1^). Under the analyzed conditions, one enzymatic activity unit was defined as the amount of enzyme that produced 1 mmol of phenylhydrazone pyruvate per minute. The specific activity was established as units per milligram (U mg^−1^) of protein used in the experiment.

The nicotinamide adenine dinucleotide (NAD^+^)-dependent isocitrate dehydrogenase (IDH; EC 1.1.1.41) activity was determined in the assay as nicotinamide adenine dinucleotide, reduced form (NADH) formation (Lin and Mcalister-Henn, 2002). The reaction was initiated with the addition of the protein extract. The product formation was determined at 340 nm, and 1 unit of enzymatic activity was defined as the formation of 1 mmol of NADH per minute under the experimental conditions. The specific activity was established as units per milligram (U mg^−1^) of protein used.

The NADP^+^-dependent isocitrate dehydrogenase (IDHP; EC 1.1.1.42) activity was determined in the assay as NADPH formation (Keys and Mcalister-Henn, 1990). The reaction was initiated with the addition of the protein extract. The product formation was determined at 340 nm, defining 1 unit of enzymatic activity as the formation of 1 mmol of NADPH per minute under experimental conditions. The specific activity was established as (U mg^−1^) of protein.

### *In silico* Analysis of the DNA-Binding Consensus for the PacC Transcriptional Regulator

Bioinformatics data mining in the genome of *T. interdigitale* H6 was performed while searching for the binding site for PacC in the promoter region of the evaluated genes. We analyzed 1,000 nucleotides upstream of the start codon to locate the consensus for PacC protein, described as GCCA(A/G)G, and performed the same for the reverse and complementary sequence C(C/T)TGGC (Tilburn et al., 1995; Espeso and Penalva, 1996).

## Results

### Modulation of the Extracellular pH and Mycelial Growth

**Figure 1:**
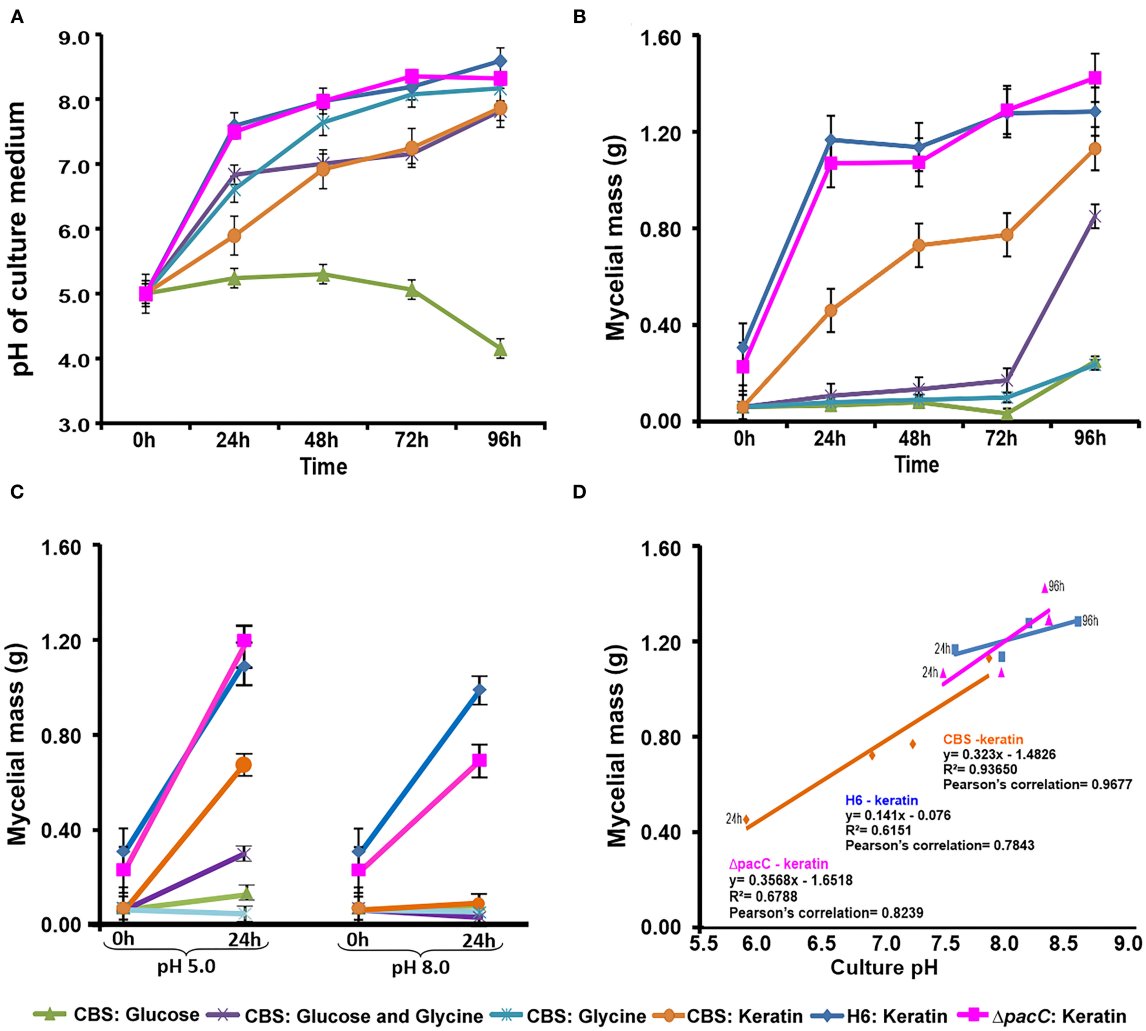
Time-dependent correlation between culture pH and mycelial mass. **(A)** Culture pH in unbuffered media (initial pH: 5.0). **(B)** Mycelial mass production in unbuffered media. **(C)** Mycelial mass production in buffered media. **(D)** Mycelial mass production and culture pH correlations. CBS, *Trichophyton rubrum* CBS 118892; H6, wild-type *Trichophyton interdigitale*; Δ*pacC, Trichophyton interdigitale* H6 strain carrying a disrupted *pacC* gene.

When *Trichophyton* species were cultured in unbuffered MM, the extracellular pH shifted from acidic to alkaline except in the case of glucose, in which the pH remained acidic during 96 h of culture (Figure 1A). A similar accumulation in mycelial mass was observed for *T. rubrum* CBS 118892 in both glycine and glucose during 96 h of cultivation. Notably, the combination of these nutrient sources enabled further accumulation of fungal mass after 72 h (glycine plus glucose, in purple) (Figure 1B). The mycelial mass developed during growth in keratin increased over time in all strains; the mycelial mass accumulation occurred independently of the *pacC* gene in *T. interdigitale* H6.

In buffered media (Figure 1C), alkaline pH suppressed the mycelial mass accumulation to a greater extent than in acidic pH. Only the growth of *T. interdigitale* H6 cultured in keratin was not inhibited by the alkaline conditions, independently of *pacC* expression. *T. rubrum* CBS 118892 showed a lower growth rate than that of *T. interdigitale* H6 in keratin at acidic pH, and the growth of *T. rubrum* CBS 118892 was inhibited in buffered (pH 8.0) medium (Figure 1D).

### *In silico* Analysis of the PacC DNA-Binding Domain

Analyses of the promoter region revealed the PacC consensus in the *sub5, icl*, and *meicl* genes among the evaluated genes (*idh1, idh2, icl, meicl, mad2, mad2B, sub3*, and *sub5*). The sequencing quality of the *idhp* gene promoter region did not allow the analysis of 1,000 upstream base pairs.

### Expression Profile of Genes Involved in Metabolic Pathways

**Figure 2:**
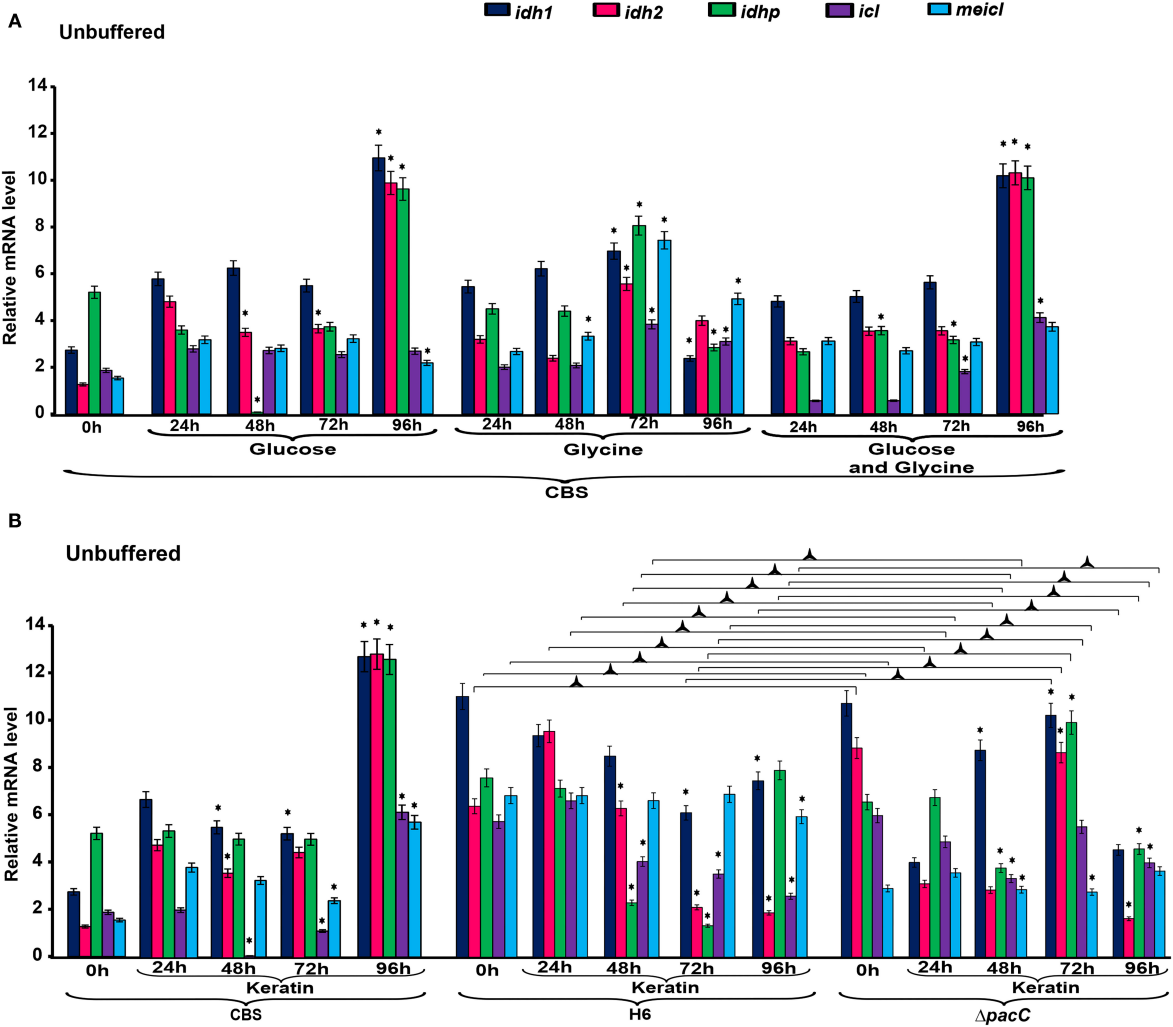
Differential expression of genes involved in metabolism under unbuffered conditions. **(A)** *T. rubrum* CBS 118892 grown in glucose, glycine or glucose plus glycine. **(B)** *T. rubrum* CBS 118892 and T. interdigitale strains grown in keratin medium. Expression of *idh1, idh2, idhp, icl*, and *meicl* genes was analyzed by RT-qPCR. The 0 h time point corresponds to 96 h cultures in Sabouraud dextrose broth. Significant differences (*P* < 0.05) in gene expression compared to that at 24 h for each nutritional source are indicated by *. The condition associated with the lowest expression was chosen as a reference for relative expression (*idhp*/*T. rubrum* in minimal medium containing glucose at 48 h). CBS, *Trichophyton rubrum* CBS 118892; H6, wild-type *Trichophyton interdigitale*; Δ*pacC, Trichophyton interdigitale* H6 strain carrying a disrupted *pacC* gene.

The expression levels of genes associated with the tricarboxylic acid (TCA) cycle, *idh1, idh2*, and *idhp*, in *T. rubrum* CBS 118892 were relatively higher in 96 h cultures in unbuffered MM in the presence of glucose, and glucose plus glycine (Figure 2A). However, in glycine, these genes were upregulated after 72 h of cultivation. A concomitant increase in the expression levels of methylisocitrate lyase (*meicl*) and isocitrate lyase (*icl*) genes was observed at 72 h that lasted until 96 h.

**Figure 3:**
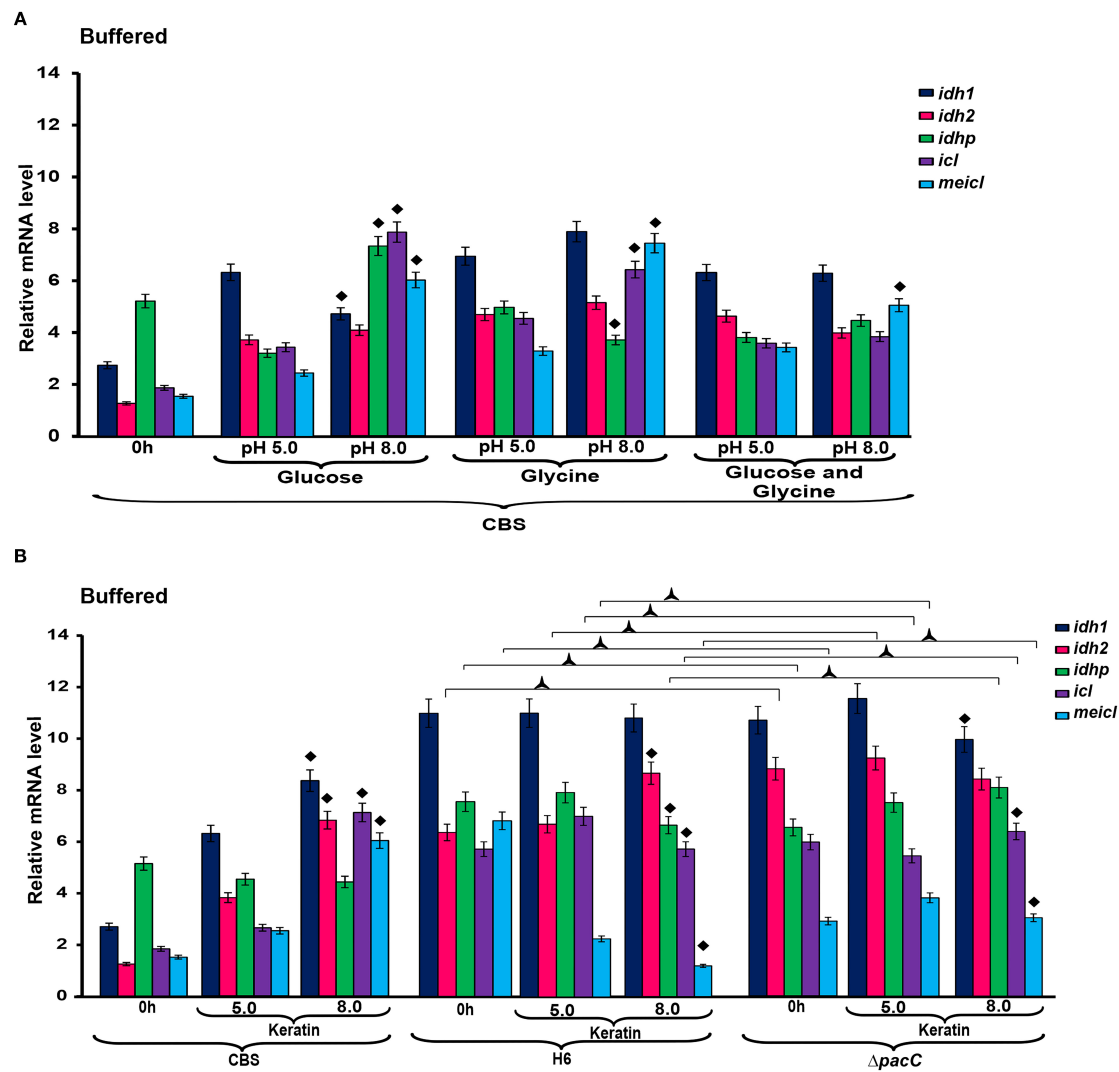
Differential expression of genes involved in metabolism under buffered conditions. **(A)** *T. rubrum* CBS 118892 grown in glucose, glycine or glucose plus glycine. **(B)** *T. rubrum* CBS 118892 and T. interdigitale strains grown in keratin medium. Expression of *idh1, idh2, idhp, icl*, and *meicl* genes was analyzed by RT-qPCR. The 0 h time point corresponds to 96 h cultures in Sabouraud dextrose broth. Significant differences (*P* < 0.05) in gene expression compared to that at 24 h of each nutritional source are indicated as ♦ for pH 8.0 compared to pH 5.0 of the respective nutritional source; Δ*pacC* strain compared to wild-type *Trichophyton interdigitale* H6 in the same condition is indicated as 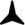. The condition associated with the lowest expression was chosen as a reference for relative expression (*idhp*/*T. rubrum* in minimal medium containing glucose at 48 h). CBS, *Trichophyton rubrum* CBS 118892; H6, wild-type *Trichophyton interdigitale*; Δ*pacC, Trichophyton interdigitale* H6 strain carrying a disrupted *pacC* gene.

The expression of *idh1, idh2*, and *idhp* was induced in *T. rubrum* CBS 118892 cultivated in keratin-containing medium after 96 h of cultivation (Figure 2B). The deletion of *pacC* upregulated the same TCA cycle genes at 72 h, presenting a different pattern from that of the wild-type strain *T. interdigitale* H6. Under buffered conditions (Figure 3A), *T. rubrum* CBS 118892 gene expression was less affected during growth in glucose plus glycine compared with that in other evaluated conditions. The deletion of *pacC* directly influenced the TCA cycle gene expression: keratin exposure (pH 8.0) induced *idh2* and *idhp* expression in the wild-type *T. interdigitale* H6 strain and induced *idh1* expression in the *T. interdigitale* mutant Δ*pacC* strain (Figure 3B).

### Intracellular Enzyme Activity

Analysis of the intracellular enzyme activity, such as the TCA cycle components IDH and IDHP, GC component ICL, and methylcitratecycle (MC) component MeICL (Table 2) revealed that ICL in *T. rubrum* CBS 118892 and *T. interdigitale* Δ*pacC* strains showed the highest specific activity in SDB medium. In contrast, the highest IDH activity was observed in *T. interdigitale* wild-type H6, when cultured in keratin. ICL activity was the highest in SDB compared with that in other cultivation conditions in *T. rubrum* CBS 118892*, T. interdigitale* H6, and in the *T. interdigitale* Δ*pacC* strains. *T. rubrum* CBS 118892 cultured in glucose plus glycine showed a high IDH and IDHP activities at 96 h.

**Table 2:** Effect of the nutritional source on metabolic enzyme activity.

	**Nutritional source**	**Final culture pH**	**IDH**	**IDHP**	**ICL**	**MeICL**
CBS	Sabouraud	6.30 ± 0.36	0.014 ± 0.004	ND	0.168 ± 0.015	0.143 ± 0.010
	Glucose (24 h)	5.24 ± 0.13	0.918 ± 0.131	1.868 ± 0.220	0.005 ± 0.001	0.056 ± 0.004
	Glucose (96 h)	4.15 ± 0.9	ND	5.351 ± 0.917*	0.084 ± 0.010*	0.120 ± 0.009*
	Glycine (24 h)	6.61 ± 0.24	0.015 ± 0.002	0.725 ± 0.113	0.018 ± 0.004	0.086 ± 0.007
	Glycine (96 h)	8.17 ± 0.11	0.147 ± 0.018*	4.931 ± 0.321*	0.040 ± 0.005*	0.045 ± 0.006*
	Glucose plus glycine (24 h)	6.83 ± 0.56	0.142 ± 0.009	0.115 ± 0.018	0.003 ± 0.001	0.020 ± 0.002
	Glucose plus glycine (96 h)	7.82 ± 0.11	18.895 ±1.358*	9.040 ± 0.544*	0.138 ± 0.008*	0.207 ± 0.012*
	Keratin (24 h)	5.89 ± 0.42	0.331 ± 0.075	0.406 ± 0.078	0.024 ± 0.005	0.097 ± 0.007
	Keratin (96 h)	7.87 ± 0.11	ND	0.531 ± 0.058	0.015 ± 0.003	0.026 ± 0.003*
H6	Sabouraud	7.84 ± 0.01	0.470 ± 0.028	0.059 ± 0.006	0.079 ± 0.006	0.070 ± 0.005
	Keratin (24 h)	7.59 ± 0.14	0.013 ± 0.004	0.443 ± 0.013	0.002 ± 0.001	0.011 ± 0.002
	Keratin (96 h)	8.59 ± 0.19	0.032 ± 0.005*	1.036 ± 0.062*	0.040 ± 0.003*	0.052 ± 0.009*
Δ*pacC*	Sabouraud	7.25 ± 0.03	0.301 ± 0.015 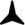	0.042 ± 0.008	0.335 ± 0.043 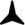	0.184 ± 0.001 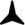
	Keratin (24 h)	7.49 ± 0.07	0.222 ± 0.046 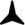	0.120 ± 0.029 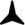	0.009 ± 0.002 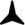	0.035 ± 0.004 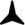
	Keratin (96 h)	8.32 ± 0.01	ND	0.425 ± 0.060* 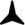	0.080± 0.009* 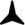	0.063 ± 0.008*

*Activities are given in U·mg^−1^. IDH, NAD^+^-isocitrate dehydrogenase; IDHP, NADP^+^-isocitrate dehydrogenase; ICL, Isocitrate lyase; MeICL, Metylisocitrate lyase; ND, not detected under the experimental conditions*.

*
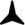
Statistical differences in Trichophyton interdigitale H6 mutant ΔpacC activity compared to the wild-type Trichophyton interdigitale H6 in the same condition (P <0.05)*.

*Statistical differencesin 96 h activity compared to 24 h in the same nutritional condition.

*CBS, Trichophyton rubrum CBS 118892; H6, wild-type Trichophyton interdigitale; ΔpacC, Trichophyton interdigitale H6 strain carrying a disrupted pacC gene*.

*Trichophyton interdigitale* wild-type H6 strain showed high IDHP activity when cultured in keratin. This activity was dependent on the *pacC* gene. At 24 h, IDHP activity in *T. interdigitale* H6 strain was similar to that in *T. rubrum* CBS 118892; however, wild-type *T. interdigitale* H6 showed an increased IDHP activity compared with that of *T. rubrum* CBS 118892 at 96 h. The *T. interdigitale* Δ*pacC* strain showed higher IDH and ICL activities than those of the wild-type *T. interdigitale* H6 strain.

We investigated the potential effect of phosphorylation on ICL activity (Table 3). An increase in ICL activity was observed in dephosphorylated protein extracts. Although in the untreated samples, ICL activity was higher in the *T. interdigitale* Δ*pacC* mutant than that in the wild-type *T. interdigitale* H6 strain, no significant difference was observed in the activity of treated protein extracts.

**Table 3:** ICL activity before and after *in vitro* dephosphorylation.

	**Nutritional**	**Untreated**	**Treated**	**Ratio**
	**source (96 h)**			**untreated:treated**
CBS	Glucose	0.084 ± 0.010	0.159 ± 0.019*	1:1.9
	Glycine	0.040 ± 0.005	0.088 ± 0.010*	1:2.2
	Glucose plus glycine	0.138 ± 0.008	0.487 ± 0.034*	1:3.5
	Keratin	0.015 ± 0.003	0.064 ± 0.012*	1:4.3
H6	Keratin	0.040 ± 0.003	0.102 ± 0.015*	1:2.5
Δ*pacC*	Keratin	0.080 ± 0.009 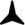	0.129 ± 0.029*	1:1.6

*Activities are given in U·mg−1. ICL, Isocitrate lyase*.

*
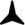
Statistical differences in Trichophyton interdigitale H6 mutant ΔpacC activity compared to the wild-type Trichophyton interdigitale H6 in the same condition (p < 0.05)*.

*Statistical differencesin treated compared to untreated in the same condition (p <0.05).

*CBS, Trichophyton rubrum CBS 118892; H6, wild-type Trichophyton interdigitale; ΔpacC, Trichophyton interdigitale H6 strain carrying a disrupted pacC gene*.

**Figure 4:**
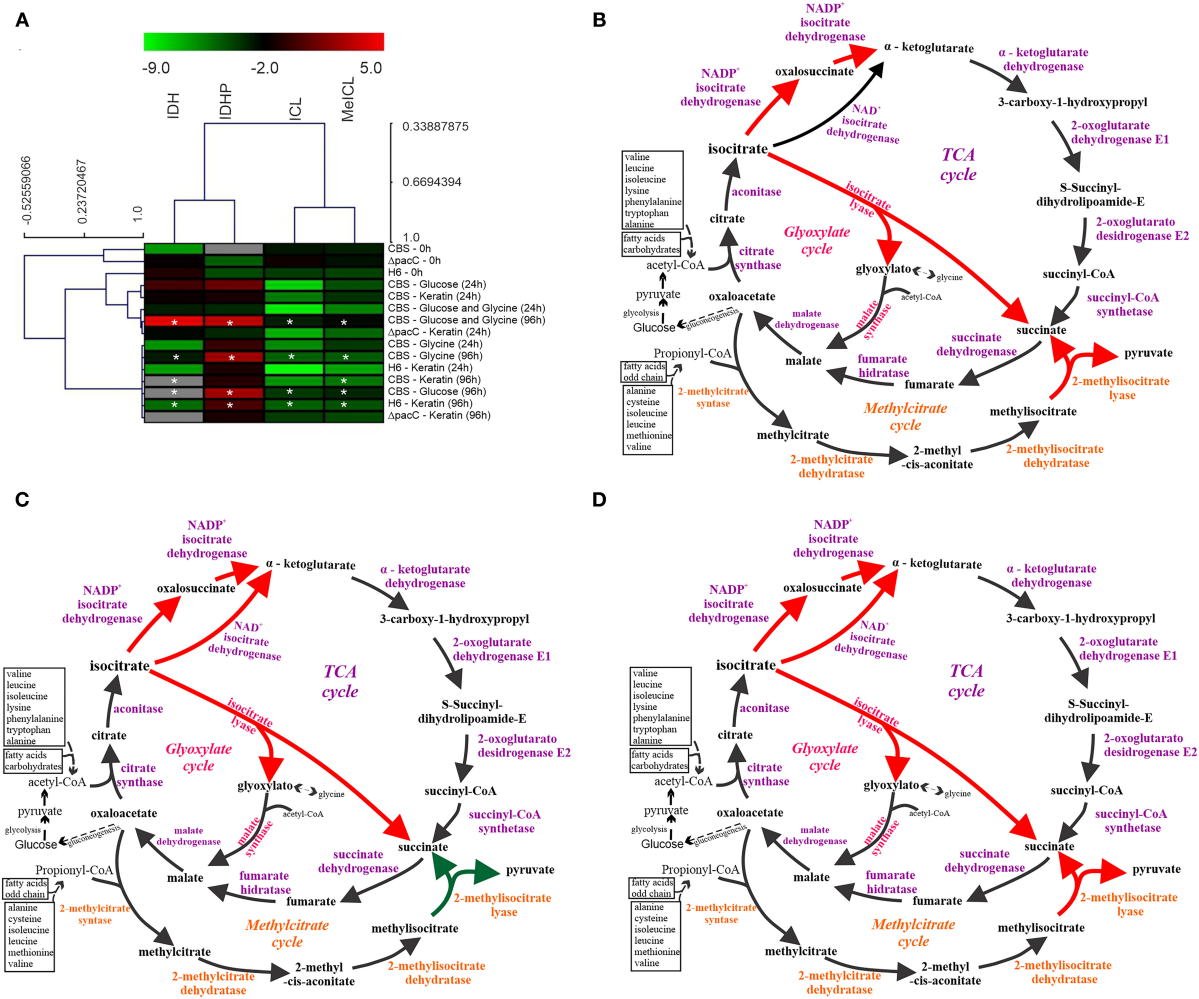
Schematic representation of TCA, GC, and MC integration in response to nutritional source in *Trichophyton* spp. **(A)** Heat map of the enzymatic activities represented as values in log2. Hierarchical clustering was performed using Pearson correlation. In gray, no enzymatic activity detected. Values are color-coded according to the top color bar. Asterisks (*) indicate significant differences in 96 h activity compared to that at 24 h in the same nutritional condition. **(B)** Schematic representation of the metabolic activity of *Trichophyton rubrum* grown in glucose and of the *Trichophyton interdigitale* H6 mutant Δ*pacC* strain grown in keratin. **(C)** *T. rubrum* grown in glycine. **(D)** *T. rubrum* grown in glucose plus glycine, and wild-type *T. interdigitale* H6 grown in keratin. Enzymes marked in orange are exclusive of the MC and those exclusive of GC are marked in pink. Dashed black arrows indicate routes that have been simplified. Red arrows indicate higher enzyme activity than that marked using green arrows (according to the heatmap). TCA, tricarboxylic acid cycle; GC, glyoxylate cycle; MC, methylcitrate cycle; CBS, *Trichophyton rubrum* CBS 118892; H6, wild-type *Trichophyton interdigitale*; Δ*pacC, Trichophyton interdigitale* H6 strain carrying a disrupted *pacC* gene.

The enzymatic activity presented in a heat map (Figure 4A) highlights two groups, one with the TCA enzymes IDH and IDHP, and another with the enzymes from anaplerotic pathways (ICL from GC, and MeICL from MC). The enzymatic activity of *T. rubrum* CBS 118892 grown in glucose, comparing 96 h with the 24 h time points, is presented in Figure 4B. This profile was also observed for the *T. interdigitale* mutant Δ*pacC* grown in keratin. The enzymatic activity in *T. rubrum* CBS 118892 in response to glycine and to glucose plus glycine is presented in Figures 4C,D, respectively. This late profile was similar to that of the enzymatic activity in wild-type *T. interdigitale* H6 in response to keratin.

### Transcriptional Profile of Genes Involved in Mitosis Control

**Figure 5:**
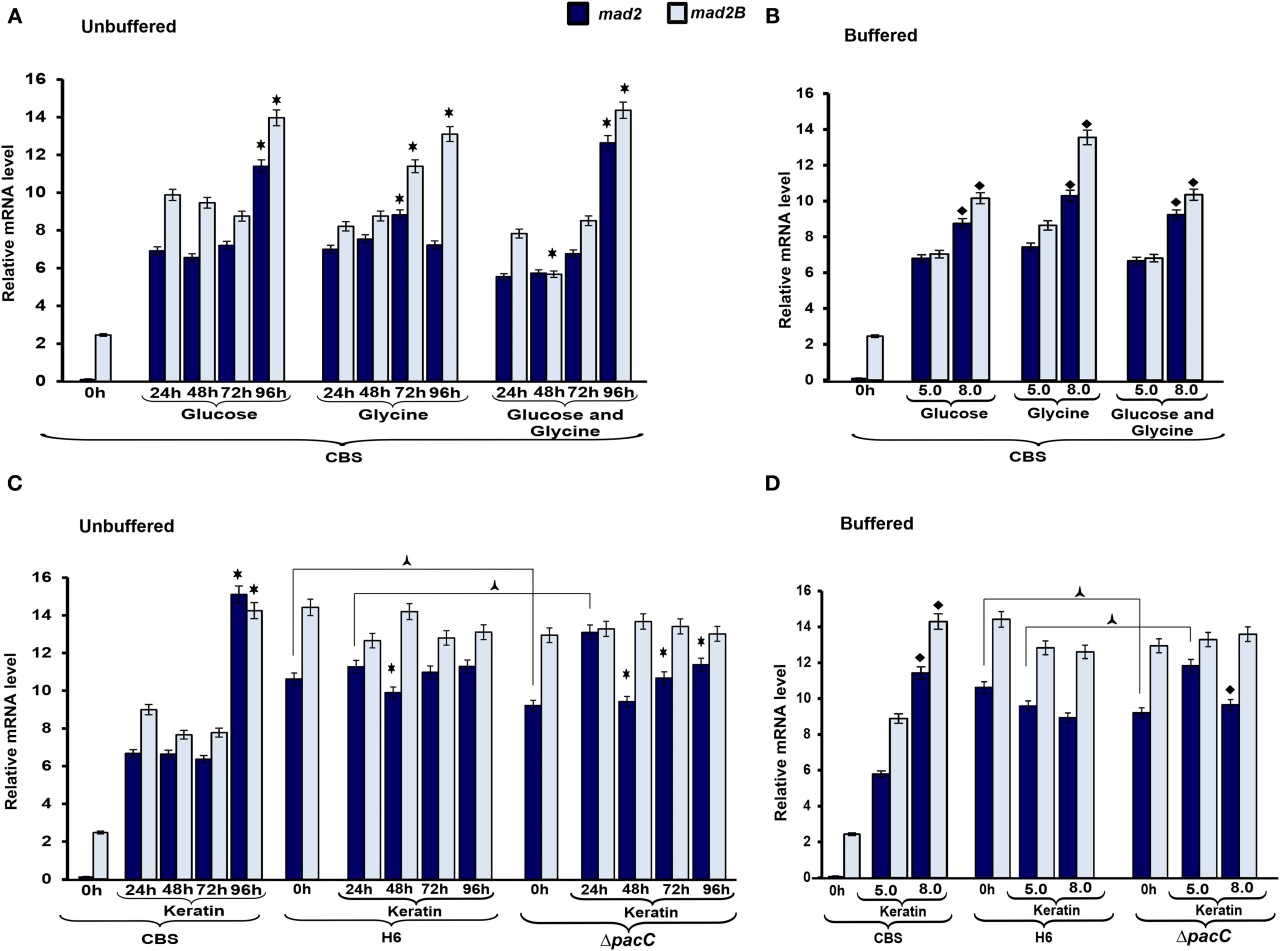
Expression profile of genes associated with cell cycle control. Expression of the genes *mad2* and *mad2B* was analyzed during growth in unbuffered **(A,C)** and buffered **(B,D)** culture media. The 0 h condition corresponds to mycelia from 96 h Sabouraud dextrose broth cultures. Data represent median values from three independent experiments. Significant differences (*P* < 0.05) in gene expression were compared to that at 24 h of each nutritional source (*A,C), pH 8.0 compared to pH 5.0 of the respective nutritional source **(**♦**B,D)**, *Trichophyton interdigitale* H6 mutant Δ*pacC* strain compared to wild-type *T. interdigitale* H6 in the respective conditions **(**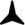 **C**,**D)**. The condition associated with the lowest expression was chosen as a reference for relative expression (*mad2*/*Trichophyton rubrum* in Sabouraud dextrose broth). CBS, *Trichophyton rubrum* CBS 118892; H6, wild-type *Trichophyton interdigitale*; Δ*pacC, Trichophyton interdigitale* H6 strain carrying a disrupted *pacC* gene.

To evaluate the mechanisms *via* which nutritional source and pH influence cell cycle-associated genes in *Trichophyton* species, we assessed the transcriptional profile of *mad2* and *mad2B* genes. The gene *mad2B* was upregulated in *T. rubrum* CBS 118892 at 96 h in the unbuffered media. The gene *mad2* was similarly upregulated in *T. rubrum* CBS 118892 at 96 h except in response to glycine, in which an earlier transcript accumulation at 72 h was observed (Figure 5A). Furthermore, both genes were upregulated in buffered-medium (pH 8.0), suggesting an expression in response to pH in *T. rubrum* CBS 118892 (Figure 5B). In unbuffered medium, *mad2* in the wild-type *T. interdigitale* H6 was downregulated at 48 h, as observed in the *T. interdigitale* mutant Δ*pacC* strain. However, the downregulation was prolonged in the mutant strain to a different extent from that in the wild-type *T. interdigitale* H6 strain (Figure 5C). The transcriptional profile observed in the buffered medium also highlights the effects of *pac*C on the *mad2* gene (Figure 5D).

### Transcriptional Profile of Endoproteases

**Figure 6:**
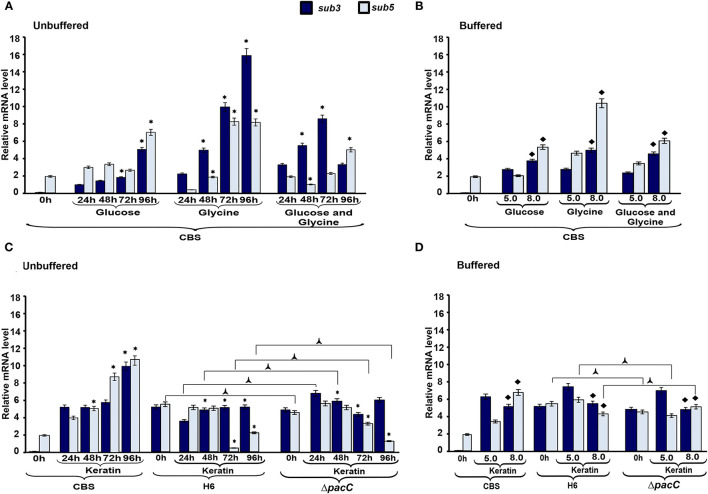
Expression profile of protease genes. The expression of *sub3* and *sub5* genes in *Trichophyton rubrum* CBS 118892 and *Trichophyton interdigitale* H6 was analyzed during growth in unbuffered **(A,C)** and buffered **(C,D)** culture medium. Time 0 h indicates Sabouraud dextrose broth cultures at 96 h. Data represent median values from three independent experiments. Significant differences (*P* < 0.05) in gene expression were compared to that at 24 h of each nutritional source (*A,**C)**, pH 8.0 compared to pH 5.0 of the respective nutritional source **(**♦**B,D)**, and *T. interdigitale* H6 mutant Δ*pacC* compared to wild-type *T. interdigitale* H6 in the respective conditions **(**
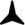
**C,D)**. The condition associated with the lowest expression was chosen as a reference for relative expression (*sub3*/*T. rubrum* in Sabouraud dextrose broth). CBS, *Trichophyton rubrum* CBS 118892; H6, wild-type *Trichophyton interdigitale*; Δ*pacC, Trichophyton interdigitale* H6 strain carrying a disrupted *pacC* gene.

To evaluate the mechanisms *via* which nutritional source and pH influence protease production in *Trichophyton* species, we assessed the transcriptional profile of subtilisin endoproteases encoded by *sub3* and *sub5*. The overall profile in *T. rubrum* CBS 118892 reveals the regulation of *sub3* and *sub5* genes over time in response to the culture media (Figure 6). In response to pH, the transcripts of both genes accumulated at pH 8.0 in *T. rubrum* CBS 118892, except for *sub3* at pH 5.0 in response to keratin. Moreover, the wild-type *T. interdigitale* H6 inversely repressed the transcriptional accumulation of *sub5*, compared with that in *T. rubrum* CBS 118892. Although also repressed, the *T. interdigitale* Δ*pacC* strain showed a considerably higher repression at 96 h, whereas the wild-type *T. interdigitale* H6 strain showed a higher repression at 72 h (Figure 6C). Both *T. interdigitale* wild-type H6 and the mutant *T. interdigitale* Δ*pacC* strains were responsive to buffered pH.

## Discussion

Fungi contain advanced complex signal transduction networks allowing their adaptation to pH fluctuation, triggering the expression of several genes to facilitate their survival in a broad range of environmental conditions (Rossi et al., 2013; Martins et al., 2019a,b). The pH shift and nutritional source are of great importance in maintaining dermatophyte growth (Martinez-Rossi et al., 2017). The hydrolysis of skin proteins releases amino acids, such as glycine, promoting the modulation of the extracellular pH from acidic to alkaline conditions. This contributes to host tissue damage and favors dermatophyte colonization (Maranhão et al., 2007; Silveira et al., 2010). However, data on the molecular aspects of nutrient acquisition and cellular growth in dermatophytes remain scarce.

Here, we evaluated pH modulation in response to nutrient variation in two *Trichophyton* species*, T. rubrum* CBS 118892 and *T. interdigitale* H6, and in the *T. interdigitale pacC* mutant strain. It is well-documented that these pathogens' *in vitro* growth depends on the initial culture pH. Our results show the correlation between the nutritional source and pH variation in fungus development. In *T.rubrum* CBS 118892, the alkalinization was glucose-repressible, as previously observed for *T. interdigitale* H6 (Maranhão et al., 2007; Mendes et al., 2012) and *Candida albicans* (Vylkova et al., 2011). This trend occurs independently of *pacC* (Mendes et al., 2012). Furthermore, the extracellular alkalinization and growth of the wild-type *T. interdigitale* H6 were not affected by the disruption of the *pacC* gene during growth in keratin as a nutritional source, as previously reported (Silveira et al., 2010).

The nutrient source influenced the growth rate of *T. rubrum* CBS 118892 in buffered conditions. After shifting from SDB to keratin-containing culture media, *T. rubrum* CBS 118892 mycelial mass accumulation was highly impacted by the buffered (pH 8.0) condition (Figure 1C). At 24 h, the pH 8.0 media inhibited *T. rubrum* CBS 118892 growth compared with the growth in unbuffered media at the same time point (Figures 1B,C). Conversely, the mycelial mass accumulated in buffered pH 5.0 was higher than that in the unbuffered media at 24 h. In this buffered condition, the mycelial mass was similar to the mass observed at 48 h, reflecting a good condition for *T. rubrum* CBS 118892 mycelial mass accumulation.

In unbuffered media, both glucose and glycine impaired *T. rubrum* CBS 118892 mycelial mass accumulation over time. The association of glucose plus glycine induced mycelial mass accumulation only after 72 h of cultivation, suggesting a late adaptation to the environment created by the combination of sugar and amino acid. *T. interdigitale* H6 mycelial mass was slightly affected comparing keratin-containing media buffered in pH 5.0 and pH 8.0. The growth profile was not affected in buffered culture media compared with that in the unbuffered keratin-containing media at 24 h; this indicated that independent of buffering the pH in culture media, *T. interdigitale* H6 development was not impaired. In contrast, *T. rubrum* CBS 118892 was more sensitive to pH fluctuation than the *T. interdigitale* H6 strain during growth in keratin, reflecting a different adaptation process between species. Overall, a positive correlation was observed between pH and mycelial mass, indicating the influence of extracellular pH in dermatophyte growth.

To determine the influence of the nutritional conditions and pH variation on gene expression in the two *Trichophyton* species, we analyzed the transcriptional profiles of genes involved in the TCA cycle (*idh1, idh2*, and *idhp*), GC (*icl*), MC (*meicl*), cell cycle control (*mad2* and *mad2B*), and keratin degradation (*sub3* and *sub5*). In *T. rubrum* CBS 118892, under unbuffered conditions, the TCA-related genes *idh1, idh2*, and *idhp* were induced at 96 h, with a slightly lower induction observed in glycine than that in glucose and glucose plus glycine (Figure 2). An increased regulation of TCA-related genes was observed compared with the regulation of GC and MC genes *icl* and *meicl*. Both the TCA cycle-related IDHs and GC-related ICL enzymes are dependent on isocitrate as a substrate. IDH enzymes oxidatively decarboxylate isocitrate into α-ketoglutarate, whereas ICL forms glyoxylate and succinate in the glyoxysomes (Kornberg, 1966). MeICL catalyzes the formation of succinate and pyruvate *via* the cleavage of 2-methylisocitrate in the last reaction of the MC (Luttik et al., 2000). Our results suggest a preference in *T. rubrum* CBS 118892 for TCA cycle activation instead of a bypass to the GC supporting the fate of isocitrate. In the presence of glycine, the transcript accumulation observed at 72 h shows the TCA cycle activation together with MeICL gene expression, suggesting the combined activation of the anaplerotic pathway. This induction persisted until 96 h. This pattern was observed at 96 h of cultivation in keratin-containing media.

In the presence of keratin under unbuffered conditions (Figure 2B), *T. interdigitale* H6 showed a *pacC*-dependent activation of the TCA-related genes at 72 h. The regulation of TCA cycle genes occurred only at 96 h. The lack of transcription factor induced an earlier activation of TCA modulation compared with that in the wild-type *T. interdigitale* H6 strain. The *icl* and *meicl* genes showed an opposite pattern of modulation in unbuffered keratin media at 72 and 96 h of cultivation: at 72 h, *icl* was repressed whereas *meicl* was not differentially expressed in the wild-type *T. interdigitale* H6 strain. In the same condition, *icl* was not differentially expressed, whereas *meicl* was repressed in the *pacC* mutant strain of *T. interdigitale* H6. The PacC DNA-binding consensus sequence in the promoter region of both genes indicated a PacC-dependent regulation. This regulation was not observed in cultures in buffered media which supports the role of pH fluctuation in *pacC* activity (Figure 3B). In buffered pH, glucose plus glycine affected gene modulation in *T. rubrum* CBS 118892 to a less extent between pH 5.0 and 8.0 conditions (Figure 3A). *T. rubrum* CBS 118892 growth in buffered keratin-containing media suggests that pH 8.0 may represent an optimal condition for metabolic activity. This pattern was not observed in *T. interdigitale* H6 (Figure 3B).

The enzymatic assays revealed an overall increased activity at 96 h compared with that at 24 h (Figures 4B–D). Only in *T. rubrum* CBS 118892, an opposite pattern was observed for the MC enzyme MeICL when grown in glycine or keratin. Moreover, PacC regulates metabolic activity, given the reduced IDHP activity and increased ICL and MeICL activities in the *T. interdigitale* H6 mutant strain, compared with those in the wild-type *T. interdigitale* H6 (Table 2). During *T. rubrum* CBS 118892 growth in glucose for 96 h, IDHP activity increased by ~3-fold, ICL activity increased by 16-fold, and MeICL activity increased by 2-fold. The activity of TCA-related enzymes increased in *T. rubrum* CBS 118892, whereas higher GC and MC activities were observed in *T. interdigitale* H6, suggesting that fungal metabolism requires anaplerotic pathways in the late growth stages. *T. rubrum* CBS 118892 growth in glucose plus glycine for 96 h was showed IDHP, ICL, and MeICL activities that were similar to the sum of the respective enzyme activities at 96 h of growth in glucose or glycine. The enzymatic profile differs from the transcript accumulation pattern observed at the 96 h time point.

One of the mechanisms to control the molecular flow between TCA and GC involves regulating the active forms of IDH and ICL *via* phosphorylation. In prokaryotes, such as *Escherichia coli*, the phosphorylation of ICL enzyme results in its activation (Robertson et al., 1988); in contrast, in eukaryotes, such as *Saccharomyces cerevisiae* (Lopez-Boado et al., 1988) and *Paracoccidioides brasiliensis* (Cruz et al., 2011), phosphorylation reduces ICL activity, leading to inactivation. In our analyses, we observed a time-dependent increase in ICL activity (Table 2). A significant difference in ICL activity was observed between *T. interdigitale* H6 mutant strain Δ*pacC* and wild-type *T. interdigitale* H6 strains cultured in keratin at 96 h. However, no significant difference was observed in ICL activity between *T. interdigitale* H6 mutant strain Δ*pacC* and the wild-type *T. interdigitale* H6 strain cultured in the same conditions after *in vitro* dephosphorylation (Table 3). These results indicate a role of the PacC transcription factor upon ICL phosphorylation. In *Aspergillus fumigatus*, phosphorylation does not affect the catalytic activity of ICL (Ebel et al., 2006). *P. brasiliensis* shows a reversible mechanism of inactivation/activation *via* phosphorylation of ICL, regulated by the carbon source, in a rapid adaptation to changes in environmental conditions (Cruz et al., 2011). This mechanism may also occur in *T. rubrum* and *T. interdigitale*.

The effects of nutrient-sensing in growth and cell cycle progression have been extensively evaluated, mainly in yeasts (Busti et al., 2010; Gutteridge et al., 2010; Yanagida et al., 2011; Alberghina et al., 2012). To evaluate the mechanisms *via* which nutritional source and pH influence cell cycle-associated genes in *Trichophyton* species, we assessed the transcriptional profile of *mad2* and *mad2B* genes. Both participate in inhibiting the anaphase-promoting complex (Chen and Fang, 2001; Bhat et al., 2015). We observed that in unbuffered conditions, the expression of both genes was induced in the latest time points evaluated (Figure 5). Buffering the media promoted the accumulation of transcripts in *T. rubrum* CBS 118892 at pH 8.0 to a different extent from that in the wild-type *T. interdigitale* H6. In *T. rubrum* CBS 118892, growth in the unbuffered medium for 96 h resulted in the upregulation of *mad2* and *mad2B*, except in glycine. In this latest condition, similar to the TCA cycle genes, the induction was observed earlier at 72 h. Under buffered (pH 8.0) conditions, *mad2* and *mad2B* expression is induced compared with that at pH 5.0. As the overall growth of *T. rubrum* CBS 118892 was inhibited at pH 8.0 (Figure 1), the gene expression profile suggests that when fungi cannot modulate environmental pH, they trigger the regulation of cell cycle control. Moreover, in *T. interdigitale*, the *pacC* deletion affected the transcription of *mad2* at pH 5.0 in buffered medium, and at pH 5.0 under unbuffered conditions, both at 24 h of cultivation (Figures 5C,D). Although the *mad2* gene does not present the consensus for PacC, our results indicate that this transcription factor is important in regulating the earlier transcription of *mad2*. The *mad2* gene has been previously considered to be differentially expressed at pH 5.0, but not at pH 8.0, based on SSH analysis after 1 h cultivation in MM supplemented with glucose, sodium nitrate, and inorganic phosphate (Silveira et al., 2010).

The most important nutritional source for the growth of dermatophytes involves keratinous substrates. The breakdown of these molecules is promoted by secretory proteases, which have specificity for different substrates (Chen et al., 2010; Tran et al., 2016). Here, we analyzed the transcript accumulation of two subtilisin endoproteases encoded by *sub3* and *sub5*. The higher expression of *sub3* and *sub5* genes in *T. rubrum* CBS 118892 was observed in glycine or keratin-containing media, in response to amino acid and protein sources (Figure 6). The metabolism of amino acids, such as glycine, released from skin proteins, results in the secretion of ammonia and shifts the ambient pH from acidic to alkaline (Maranhão et al., 2007; Silveira et al., 2010; Mendes et al., 2012; Martinez-Rossi et al., 2017). A glycine-rich environment is well-tolerated by *T. rubrum* CBS 118892, thereby enhancing *sub3* and *sub5* transcript accumulation. Moreover, the upregulation in glucose in 96 h cultures suggests that these genes may also be required for the later stages of development in a non-protein medium. Previously, the expression of genes coding for metalloproteases and subtilisins has been reported to remain relatively stable during *T. rubrum* growth in glucose, whereas they are upregulated in culture media containing collagen, elastin, keratin salt, or human skin (Leng et al., 2009). In unbuffered keratin media, the proteolytic activity of *T. rubrum* CBS 118892 increased over time, whereas the overall proteolytic profile of *T. interdigitale* H6 mutant strain Δ*pacC* showed a decreased activity over time. The same pattern was not observed in the wild-type *T. interdigitale* H6 strain reflecting PacC-associated regulation. Under buffered conditions, both *sub3* and *sub5* genes showed increased transcript accumulation in *T. rubrum* CBS 118892 under alkaline condition, except in keratin-containing media. Both *T. rubrum* CBS 118892 and *T. interdigitale* H6 repress *sub3* transcript accumulation in keratin, independently of *pacC*. The transcript accumulation of *sub5*, which presents the consensus for PacC, increased in *T. rubrum* CBS 118892 and Δ*pacC* mutant of *T. interdigitale* H6 strains; whereas, it reduced in wild-type *T. interdigitale* H6, confirming a PacC-dependent modulation.

Our data show that although dermatophytes are strikingly similar in genetic content (Martinez et al., 2012), the mechanisms for adaptation to environmental conditions and the transcriptional responses to nutritional sources and pH are distinct. Also, strains recently isolated from patients may show different responses concerning the enzymatic activity profile (Gnat et al., 2018). These differences may represent determinants for the incidence of infection with each species.

## Data Availability Statement

The original contributions presented in the study are included in the article/supplementary materials, further inquiries can be directed to the corresponding author/s.

## Author Contributions

AC and AR designed the project. AC, RS, NP, GT, and NM performed the experiments. AC, RS, MM, NP, NM-R, and AR wrote the manuscript. AR and NM-R supervised the study and prepared the manuscript. All authors participated in data analysis, in the critical revision of the manuscript, and approved the final version.

## Funding

This work was supported by research grants from the Brazilian Funding Agencies FAPESP (Proc. No. 2019/22596-9 and Fellowship Nos. 2018/11319-1 to MM, 2009/08411-4 to NP, and 2009/15426-8 to GT), CNPq (Proc. Nos. 305797/2017-4 and 304989/2017-7), CAPES (Finance Code 001), and FAEPA of the HCFMRP-USP.

## Conflict of Interest

The authors declare that the research was conducted in the absence of any commercial or financial relationships that could be construed as a potential conflict of interest.

## Publisher's Note

All claims expressed in this article are solely those of the authors and do not necessarily represent those of their affiliated organizations, or those of the publisher, the editors and the reviewers. Any product that may be evaluated in this article, or claim that may be made by its manufacturer, is not guaranteed or endorsed by the publisher.
